# Global metabolome profiling of exhaled breath condensates in male smokers with asthma COPD overlap and prediction of the disease

**DOI:** 10.1038/s41598-021-96128-7

**Published:** 2021-08-17

**Authors:** Nilanjana Ghosh, Priyanka Choudhury, Mamata Joshi, Parthasarathi Bhattacharyya, Sushmita Roychowdhury, Rintu Banerjee, Koel Chaudhury

**Affiliations:** 1 School of Medical Science and Technology, Indian Institute of Technology Kharagpur, Kharagpur, 721302 India; 2 National Facility for High-Field NMR, Tata Institute of Fundamental Research, Mumbai, India; 3 Institute of Pulmocare and Research, Kolkata, India; 4 Apollo Gleneagles Hospitals, Kolkata, India; 5 Department of Agricultural and Food Engineering, Indian Institute of Technology Kharagpur, Kharagpur, India

**Keywords:** Biological techniques, Biomarkers, Diseases

## Abstract

Asthma—chronic obstructive pulmonary disease (COPD) overlap, termed as ACO, is a complex heterogeneous disease characterised by persistent airflow limitation, which manifests features of both asthma and COPD. These patients have a worse prognosis, in terms of more frequent and severe exacerbations, more frequent symptoms, worse quality of life, increased comorbidities and a faster lung function decline. In absence of clear diagnostic or therapeutic guidelines, ACO presents as a challenge to clinicians. The present study aims to investigate whether ACO patients have a distinct exhaled breath condensate (EBC) metabolic profile in comparison to asthma and COPD. A total of 132 age and BMI matched male smokers were recruited in the exploratory phase which consisted of (i) controls = 33 (ii) asthma = 34 (iii) COPD = 30 and (iv) ACO = 35. Using nuclear magnetic resonance (NMR) metabolomics, 8 metabolites (fatty acid, propionate, isopropanol, lactate, acetone, valine, methanol and formate) were identified to be significantly dysregulated in ACO subjects when compared to both, asthma and COPD. The expression of these dysregulated metabolites were further validated in a fresh patient cohort consisting of (i) asthma = 32 (ii) COPD = 32 and (iii) ACO = 40, which exhibited a similar expression pattern. Multivariate receiver operating characteristic (ROC) curves generated using these metabolites provided a robust ACO classification model. The findings were also integrated with previously identified serum metabolites and inflammatory markers to develop a robust predictive model for differentiation of ACO. Our findings suggest that NMR metabolomics of EBC holds potential as a platform to identify robust, non-invasive biomarkers for differentiating ACO from asthma and COPD.

## Introduction

As per the definition provided by the Global Initiative for Asthma (GINA), asthma is considered to be a heterogeneous disease, with characteristics of chronic airway inflammation, clinical history of wheezing, shortness of breath, chest tightness and cough, together with reversible airflow limitation^1^. Chronic obstructive pulmonary disease (COPD), defined by the Global Initiative for Chronic Obstructive Lung Disease (GOLD), is a disease characterized by persistent respiratory symptoms like dyspnea, cough or sputum production and irreversible airflow obstruction. Emphysema and chronic bronchitis are usually seen in these patients. The changes in COPD are brought about by inhalation of noxious particles or gases and is also influenced by host factors including abnormal lung development^2,3^.

A significant proportion of older patients with chronic airflow limitation (i.e. not completely reversible after bronchodilation) have diagnosis and/or features of both asthma and COPD, particularly amongst smokers^4,5^. Such patients are termed as ‘overlap’ cases. However, there is no generally agreed term or defining features for this category^4,6,7^. Asthma and COPD are both heterogeneous diseases, with a range of underlying mechanisms. Similarly, overlap itself does not represent a single disease or a single phenotype. Gibson and Simpson proposed the term asthma COPD overlap syndrome (ACOS) in 2009 to describe patients with features of both, asthma and COPD^4^. The GINA and GOLD global bodies later replaced the term ACOS by asthma COPD overlap (ACO) as the former was often misinterpreted as a single disease entity^8^. There is general agreement that patients with features of both asthma and COPD experience a more rapid decline in lung function, have poor quality of life, experience frequent exacerbations, have increased mortality and consume more resources than asthma or COPD alone^9,10^. These patients are generally treated with inhaled corticosteroids (ICS) with add-on treatment of long-acting beta-agonist (LABA) and/or long-acting muscarinic antagonists (LAMA), whenever necessary^8^. ACO patients have been excluded from randomised controlled trials (RCTs) till date and this population is also poorly characterised in most mechanistic studies. Precise diagnosis of an ACO patient is the first step towards development of an effective treatment plan.

Metabolomics is the large-scale study of small molecules, commonly known as metabolites produced during normal endogenous metabolism within biofluids, cells, tissues or organisms. The ever expanding metabolomic approach has moved on beyond biomarker discovery to provide insight into mechanistic changes associated with various physiological conditions, including diseases^11^. Exhaled breath condensate (EBC) has emerged as a popular clinical tool over the past two decades. EBC is a non-invasive method of sampling lung epithelial lining fluid, and is obtained by cooling the exhaled air from spontaneous breathing. It is predominantly composed of water vapour along with volatile and non-volatile substances from the lower airways. Collection of EBC involves minimal technical skills and is not associated with any discomfort or risk^12^. Nuclear magnetic resonance (NMR) spectroscopy is a promising approach for the identification of metabolites in EBC with prognostic and predictive significance. Though not very sensitive, NMR is characterized by inherent distinctive advantages, which include minimal sample preparation, rapid spectra acquisition time, and the possibility to perform an untargeted analysis limited to the chemical nature of metabolites. These advantages have promoted NMR-based metabolomics of EBC to the rank of a valuable method for an efficient investigation of a variety of lung diseases^13^.

It is evidenced that electronic-nose (eNose) and NMR metabolomics of EBC can distinguish patients with respiratory disorders, including asthma and COPD^14,15^. Also, EBC metabolic profiling using NMR can be effectively used for differential diagnosis between newly diagnosed asthma and COPD^16^. It is reported that NMR spectral signatures of EBC can be used for the discovery and characterization of different asthma endotypes^17^.

A number of encouraging reports related to NMR metabolomics of EBC in asthma and COPD motivates us to test the hypothesis that this method could be a helpful tool to explore ACO. For this purpose, the ability of this technique to identify metabolic patterns that discriminate the EBC metabolome of ACO from asthma and COPD is ascertained. Our previous studies using NMR, gas chromatography–mass spectrometry (GC–MS) and immunological profiling have indicated significantly altered expression of 36 metabolites and immunological markers in serum of ACO patients as compared with asthma and COPD^18,19^. We have also attempted to integrate these markers with the present EBC metabolomic signatures for accurate prediction of ACO.

## Results

### Discovery phase

**Figure 1:**
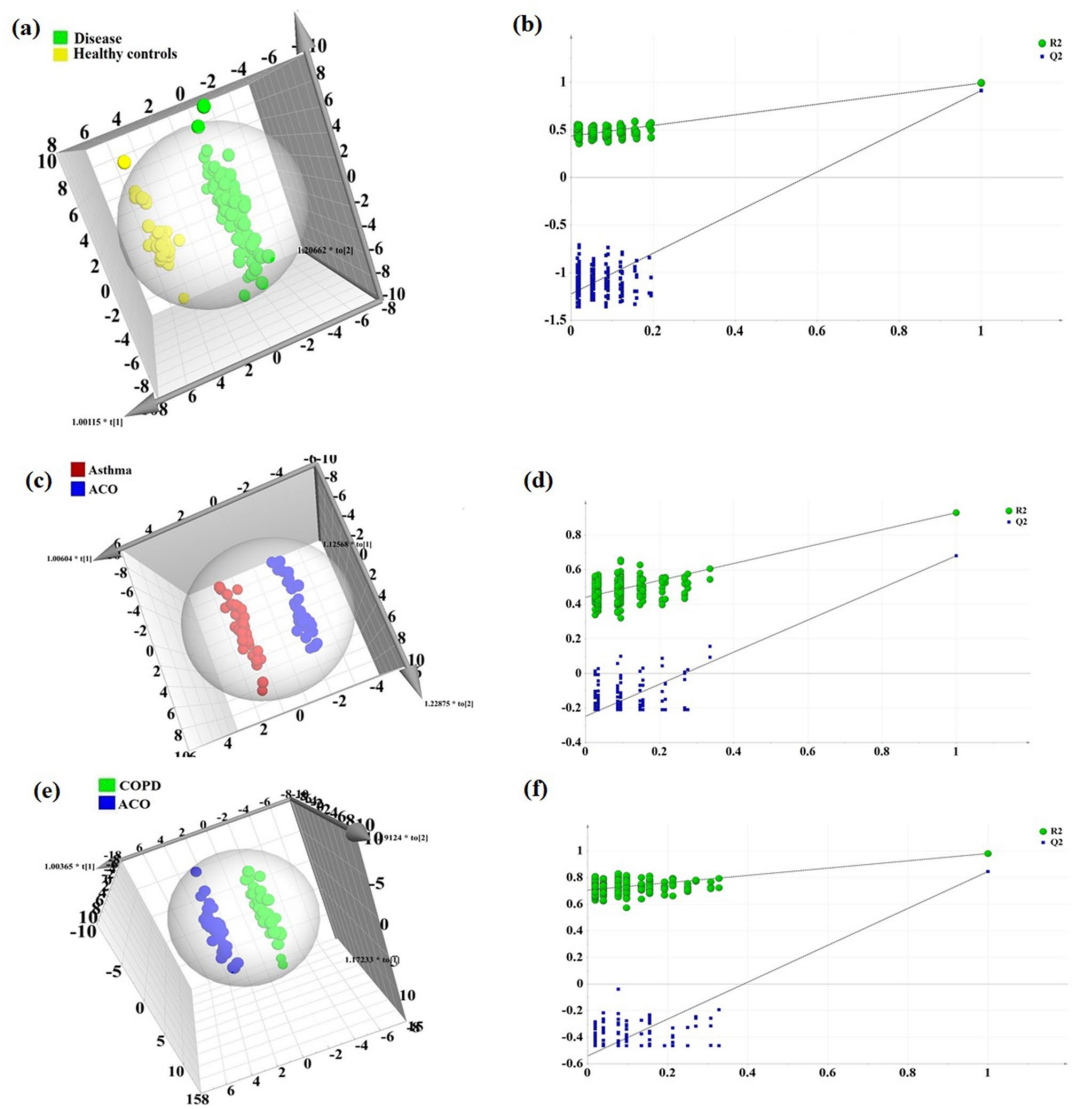
Orthogonal projections to latent structures discriminant analysis (OPLS-DA) model removes outliers which do not contribute to class separation. OPLS-DA model shows optimized discrimination between (**a**) obstructive lung diseases and healthy controls (R2Y = 0.979 and Q2 = 0.881, CV-ANOVA score *p* = 0), (**c**) ACO and asthma (R2Y = 0.982 and Q2 = 0.862, CV-ANOVA score *p* = 0) and (**e**) ACO and COPD (R2Y = 0.98 and Q2 = 0.862, CV-ANOVA score *p* = 0). Response permutation test (n = 200) to estimate the statistical significance of the PLS-DA models. (**b**) Healthy controls vs. diseases R2 = (0.0, 0.433), Q2 = (0.0, − 1.22), (**d**) ACO vs. asthma R2 = (0.0, 0.44), Q2 = (0.0, − 0.248) and (**f**) ACO vs. COPD R2 = (0.0, 0.706), Q2 = (0.0, − 0.541). All models were generated using SIMCA 13.0.2 (Umetrics, Sweden). *COPD* chronic obstructive pulmonary disease, *ACO* asthma COPD overlap.

The baseline clinical characteristics of all subjects recruited is tabulated in Table 1. For the initial part of the study, the discovery phase samples were assigned into two groups, obstructive lung diseases (asthma, COPD and ACO) and healthy controls. The supervised approach of partial least squares discriminant analysis (PLS-DA) and orthogonal projections to latent structures discriminant analysis (OPLS-DA) were applied to unit variance scaled NMR binned spectra of both the groups. PLS-DA models displayed class separation while OPLS-DA, when applied to the dataset with factors unrelated to group characters removed, resulted in improved discrimination between the two groups (R2 = 0.979 and Q2 = 0.881; analysis of variance testing of cross validated predictive residuals (CV ANOVA) score *p* = 0) (Fig. 1a). In the permutation test, R2 and Q2 values were found significantly higher than the 200 permutated models (Fig. 1b).

**Table 1:** Clinical characteristics of the recruited subjects.

	Discovery cohort					Validation cohort			
	CONTROLS	ASTHMA	COPD	ACO	*p* value	ASTHMA	COPD	ACO	*p* value
Total Number of subjects (n)	33	34	30	35		32	32	40	
Age (years)	50.90 ± 7.20	51.91 ± 7.16	54.97 ± 6.11	53.97 ± 5.89	ns	53.73 ± 5.38	55.26 ± 4.2	54.58 ± 4.6	ns
Body Mass Index (kg/m^2^)	21.35 ± 1.64	21.43 ± 1.62	20.78 ± 1.58	20.96 ± 1.65	ns	21.79 ± 1.59	19.13 ± 1.73	20.29 ± 1.22	ns
Pre BD FEV1% predicted	97.6 ± 17.8	61.3 ± 15.3	55.3 ± 14.6	52.7 ± 13.6	< 0.0001	64.4 ± 10.6	53.1 ± 12.6	50.5 ± 11.2	< 0.0001
**Post BD lung function**									
FEV1 (l)	3.8 ± 0.2	2.2 ± 0.2	1.5 ± 0.5	1.9 ± 0.2	< 0.0001	2.4 ± 0.1	1.3 ± 0.4	2.05 ± 0.2	< 0.0001
FVC (l)	4.6 ± 0.3	3.2 ± 0.8	2.9 ± 0.4	3.0 ± 0.8	< 0.0001	3.5 ± 0.2	2.7 ± 0.4	3.2 ± 0.5	< 0.0001
FEV1% predicted	99.2 ± 16.8	70.4 ± 11.3	57.6 ± 10.2	59.1 ± 9.8	< 0.0001	72.7 ± 9.7	56.9 ± 8.5	57.5 ± 6.3	< 0.0001
FEV1 /FVC (%)	83.2 ± 2.1	68.8 ± 10.1	58.5 ± 6.4	56.7 ± 2.4	< 0.0001	69.1 ± 7.2	50.5 ± 7.3	61.3 ± 7.2	< 0.0001
**Smoking status (n)**									
Former smokers	15	21	20	13		15	21	12	
Current smokers	18	13	10	22		17	11	28	
Smoking history (pack-years)	15.3 ± 5.89	16.8 ± 4.88	35.04 ± 5.6	29.9 ± 5.7	< 0.0001	14.7 ± 3.78	37.2 ± 4.9	31.9 ± 4.7	< 0.0001
Blood eosinophil cell/μl, median (IQR)	120 (0–160)	340 (0–3840)	140(0–300)	330 (0–1500)	< 0.0001	365 (0–3840)	150(0–300)	345(0–1500)	< 0.0001
Frequency of exacerbation/year	–	0.95 ± 0.20	1.78 ± 0.36	1.11 ± 0.75	< 0.0001	0.82 ± 0.11	1.87 ± 0.22	1.21 ± 0.56	< 0.0001
**Respiratory symptoms**									
Wheezing	–	17.4 ± 0.6	13.3 ± 0.7	25.3 ± 0.4	< 0.0001	19.1 ± 0.3	14.7 ± 0.4	27.6 ± 0.6	< 0.0001
Expectoration	–	12.1 ± 0.3	19.4 ± 0.3	15.6 ± 0.5	< 0.0001	10.4 ± 0.5	20.3 ± 0.6	13.8 ± 0.3	< 0.0001
mMRC scale (0–4)	–	-	2.4 ± 0.21	1.2 ± 0.6	< 0.0001	-	2.25 ± 0.51	1.35 ± 0.5	< 0.0001
Atopic status/allergy n (%)	–	29 (86)	3 (11)	24 (68)	< 0.0001	27 (83)	2 (7)	29 (72)	< 0.0001
**Treatment n (%)**									
ICS	–	33 (97)	17 (58)	27 (78)	< 0.0001	28 (89)	19 (60)	33 (82)	< 0.0001
LABA	–	25 (74)	26 (87)	29 (82)	< 0.0001	22 (70)	26 (82)	31 (78)	< 0.0001
LAMA	–	15.3 (45)	25 (83)	27 (76)	< 0.0001	10 (32)	24 (75)	28 (70)	< 0.0001
PD4I	–	-	4 (14)	3 (8)	< 0.0001	-	4 (12)	2 (5)	< 0.0001
LTRA/theophylline	–	24 (72)	1 (3)	16 (45)	< 0.0001	20 (63)	2 (5)	16 (39)	< 0.0001

Data are presented as mean ± SD or percentages, unless otherwise stated.

*COPD* chronic obstructive pulmonary disease, *ACO* asthma COPD overlap, *BD* bronchodilator, *FEV1* forced expiratory volume in 1 s, *FVC* forced vital capacity, *ICS* inhaled corticosteroids, *LABA* long-acting beta agonists, *LAMA* long-acting antimuscarinics, *PD4I* phosphodiesterase-4-inhibitior, *LTRA* leukotriene receptor antagonist. Differences between groups were assessed by using the 1-way ANOVA test with post hoc Tukey HSD. *p* < 0.05 was considered statistically significant.

Furthermore, OPLS-DA model optimized separation between ACO vs. asthma (R2 = 0.982 and Q2 = 0.862; CV ANOVA score *p* = 0) (Fig. 1c) and ACO vs. COPD (R2 = 0.98 and Q2 = 0.862; CV ANOVA score *p* = 0) (Fig. 1e). Permutation test indicated that the generated models could predict classes better than chance (Fig. 1d,f).

**Figure 2:**
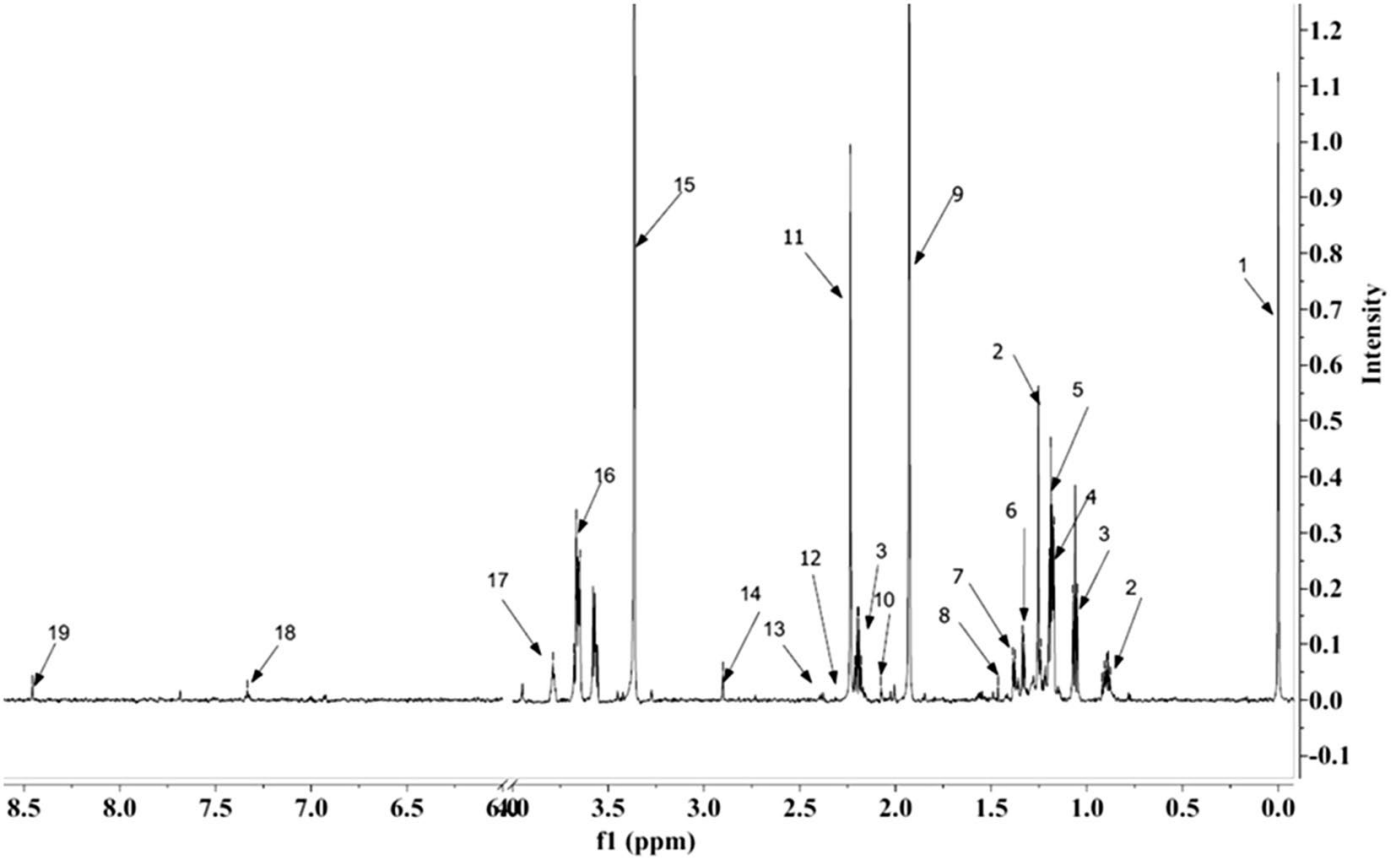
Representative 800 MHz 1H-NMR zgesgp spectra of exhaled breath condensate (EBC) collected from an ACO patient. 1.TSP 2. Fatty acid 3. Propionate 4. Isopropanol 5. Ethanol 6. Lactate 7. Threonine 8. Alanine 9. Acetate 10. Proline 11. Acetone 12. Valine 13. Pyruvate 14. Trimethylamine 15. Methanol 16. Glycerol 17. 2,3-butanediol 18. Phenylalanine 19. Formate. The NMR spectra was assigned using MestReNova version 7.1.0 (Mestrelab Research, Santiago de Compostela, Spain).

A typical representative 1H NMR EBC spectrum of an ACO patient comprising of signals arising from energy metabolites, organic acids and amino acids is shown in Fig. 2. Eighteen metabolites could be consistently identified using 2D NMR experiments, human metabolome database (HMDB) and literature. Analysis was based on these consistently identified metabolites.

Based on variable-importance in projection (VIP) ≥ 1 and S-line plot correlation value |r|≥ 0.6, highly significant variables responsible for class separation between disease and controls were identified for ACO vs asthma and ACO vs COPD. Ten bins and twelve bins, respectively qualified the screening criteria described above. Eight common bins corresponding to metabolites from both ACO vs asthma and ACO vs COPD models were considered for further analysis. These bins corresponded to the metabolites propionate, lactate, valine, fatty acid, acetone, isopropanol, formate and methanol. To cross-validate the binning approach, integral values of these metabolites were subjected to univariate analysis (UVA). Metabolites such as propionate, isopropanol and acetone were found to be up-regulated in ACO as compared to both, asthma and COPD (Table 2). Only valine was observed to be downregulated in ACO as compared to both, asthma and COPD. Fatty acid, lactate and methanol were also found to be significantly dysregulated with contrasting trends in ACO with respect to asthma and COPD. The sample peaks identified in the chemical shift region 5.1–9.0 ppm (aromatic region) were also subjected to one way ANOVA (Dunnett’s post hoc test) or Kruskal–Wallis test (Dunn’s post hoc test), as applicable and only formate level was found to be significantly dysregulated in ACO.

**Table 2:** Human Metabolome Database identifiers (HMDB ID), multivariate data analysis (variable influence on projection (VIP) scores, false discovery rate (FDR) adjusted *p* value), fold changes and pairwise univariate (ANOVA/Kruskal Wallis test) values are provided for the 8 significantly altered metabolites common to ACO vs. asthma and ACO vs. COPD.

Metabolites	Chemical shift (ppm)	HMDB ID	Discovery cohort							Validation cohort				
			VIP scores		Fold change		Significance		FDR	Fold change		Significance		FDR
			ACO vs Asthma	ACO vs COPD	ACO vs Asthma	ACO vs COPD	Pairwise *p* value			ACO vs Asthma	ACO vs COPD	Pairwise *p* value		
							ACO vs Asthma	ACO vs COPD				ACO vs Asthma	ACO vs COPD	
**Fatty acid**	0.89	–	1.41	1.31	0.81	1.26	**	*	< 0.0001	0.82	1.29	*	*	< 0.0001
**Propionate**	1.06	HMDB0000237	1.68	1.53	1.22	1.28	*	*	0.003385	1.08	1.16	ns	**	0.0025798
**Isopropanol**	1.18	HMDB0000863	2.4	1.82	1.22	1.17	***	*	0.001206	1.15	1.09	***	*	< 0.0001
**Lactate**	1.33	HMDB0000190	1.35	1.45	1.31	0.81	*	**	< 0.0001	1.94	0.64	**	***	< 0.0001
**Acetone**	2.23	HMDB0001659	1.59	1.77	1.15	1.11	***	*	< 0.0001	1.21	1.17	***	**	< 0.0001
**Valine**	2.26	HMDB0000883	1.46	1.68	0.64	0.7	***	*	< 0.0001	0.75	0.8	***	*	< 0.0001
**Methanol**	3.36	HMDB0001875	1.24	1.14	1.32	0.83	**	*	< 0.0001	1.36	0.82	***	**	< 0.0001
**Formate**	8.46	HMDB0000142	1.29	1.13	1.32	0.75	*	*	< 0.0001	1.28	0.85	***	**	< 0.0001

A new subject cohort (validation cohort) was recruited to confirm the findings of the exploratory (discovery) patient cohort.

*COPD* Chronic obstructive pulmonary disease, *ACO* Asthma COPD overlap.

*p < 0.05; **p < 0.01; ***p < 0.0001; *ns* not significant.

### Receiver operating characteristic (ROC) curve analysis

**Figure 3:**
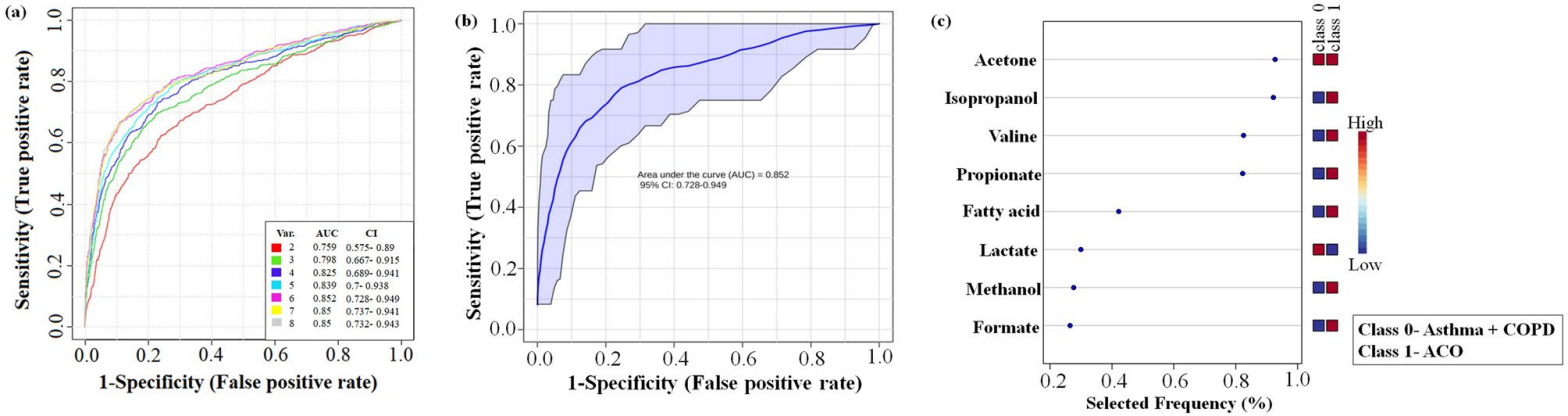
(**a**) Comparison of various receiver operating characteristic (ROC) curves using different number of variables based on Monte Carlo cross validation (MCCV) performance at 95% confidence interval. The ROC curves were generated using Metaboanalyst 4.0 (https://www.metaboanalyst.ca/). *Var.* variable, *AUC* area under curve, *CI* confidence interval. (**b**) Model 5 ROC curve generated using 6 variables shows the highest AUC = 0.852 and good power in discriminating ACO from asthma and COPD. (**c**) Significant features plot ranked by frequencies of being selected for classification derived from 6 variable multivariate receiver operating characteristic (ROC) curve.

Next, multivariate ROC curves were generated for the significantly altered 8 common metabolites of the two groups (ACO vs. asthma and ACO vs. COPD). In order to establish a predictive model that could differentiate ACO from a group of both asthma and COPD patients together, we merged the data set of asthma and COPD (referred to as 0) and ACO (referred to as 1). ROC curves were generated by Monte-Carlo cross validation (MCCV), a method involving balanced sub-sampling. In each MCCV, two thirds (2/3) of the samples were used to evaluate the feature importance. The classification models were generated using the top important features, subsequently the models were validated on the 1/3 of the samples left out. The procedure was repeated multiple times to calculate the performance and confidence interval of each model. The multivariate ROC curves based on cross validation (CV) performance are shown in Fig. 3a. The curves of all models are averaged across all CV runs. The highest AUC was obtained for model 5, generated using 6 features (Fig. 3b). The significant features ranked using mean importance measure for the model are shown in Fig. 3c. The metabolites acetone, isopropanol, valine, propionate, fatty acid and lactate show maximum importance in generation of the 5^th^ model.

### Validation phase

For validation of the 8 EBC metabolites identified in the discovery phase, UVA was performed on the data generated in the validation cohort. These metabolites viz propionate, lactate, valine, fatty acid, acetone, isopropanol, formate and methanol exhibited a similar trend yet again in the new cohort, thereby confirming our findings (Table 2). To strengthen our findings, MVA was also performed on the validation cohort and the results were found to be similar to that of the discovery cohort (“Supplementary Materials").

### Correlations and predictive modelling

**Figure 4:**
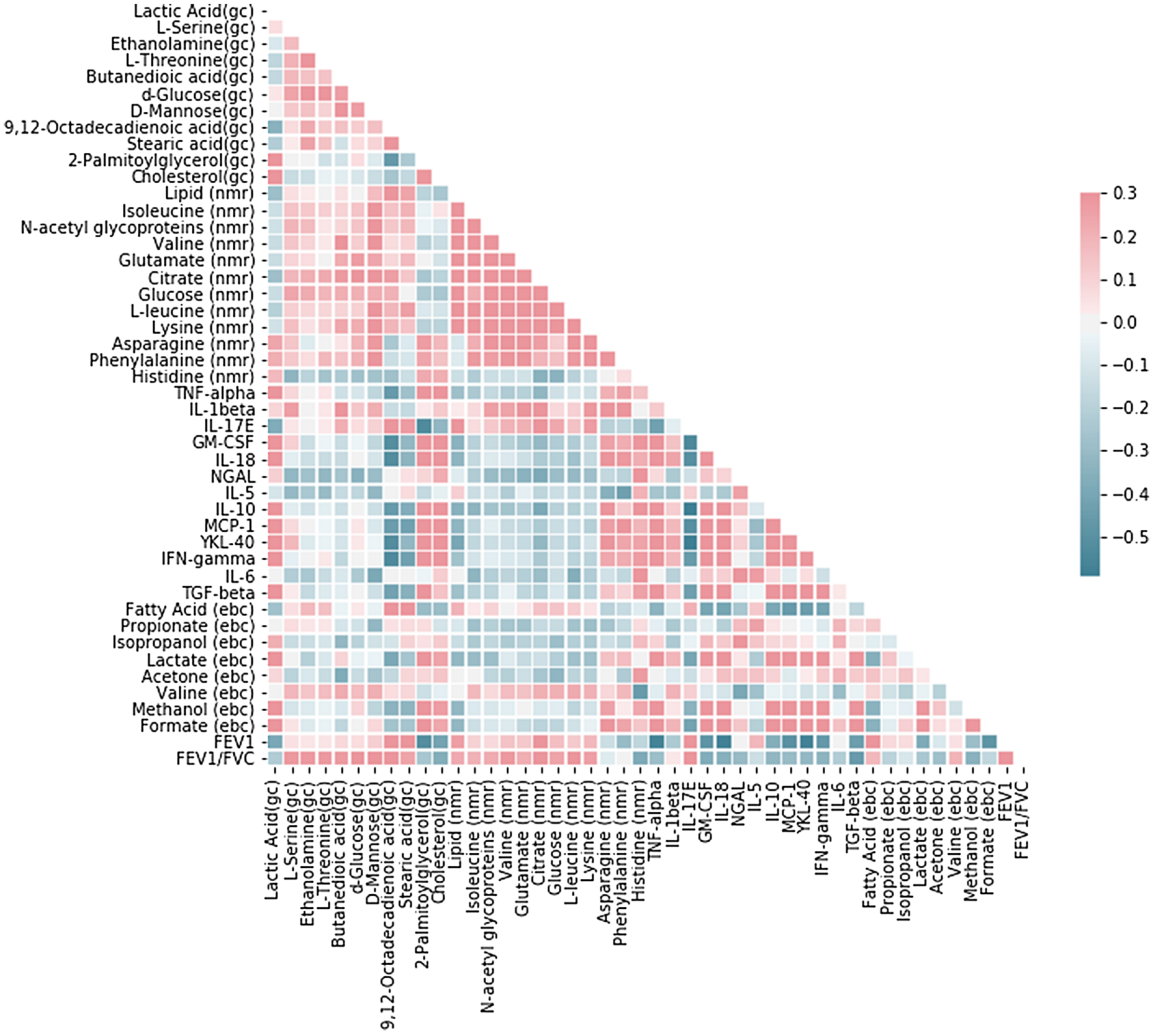
Correlation heat map of 46 significantly altered markers in ACO using Pearson’s correlation coefficients. The analysis was performed using Scikit-learn package with Python 3.8 (https://scikit-learn.org/stable). (nmr)—depicts the altered metabolites detected in serum using NMR based metabolomics, (gc)—depicts the altered metabolites detected in serum using GC MS based metabolomics, (ebc)—depicts the altered metabolites detected in EBC using NMR based metabolomics.

A combination of positive and negative correlations were observed between metabolites and immunological markers. This visualization further supports the hypothesis of tight interplay between metabolism and inflammation in ACO. Except interleukin-1beta (IL-1β) and IL-17E, the inflammatory markers exhibited negative correlation with the significantly altered metabolites. These metabolites predominantly showed a positive correlation with lung function parameters such as forced expiratory volume in one second (FEV1) and ratio of the forced expiratory volume in one second to the forced vital capacity of the lungs (FEV1/FVC). The inflammatory markers, on the other hand, mostly displayed negative correlation with lung function parameters (Fig. 4).

**Figure 5:**
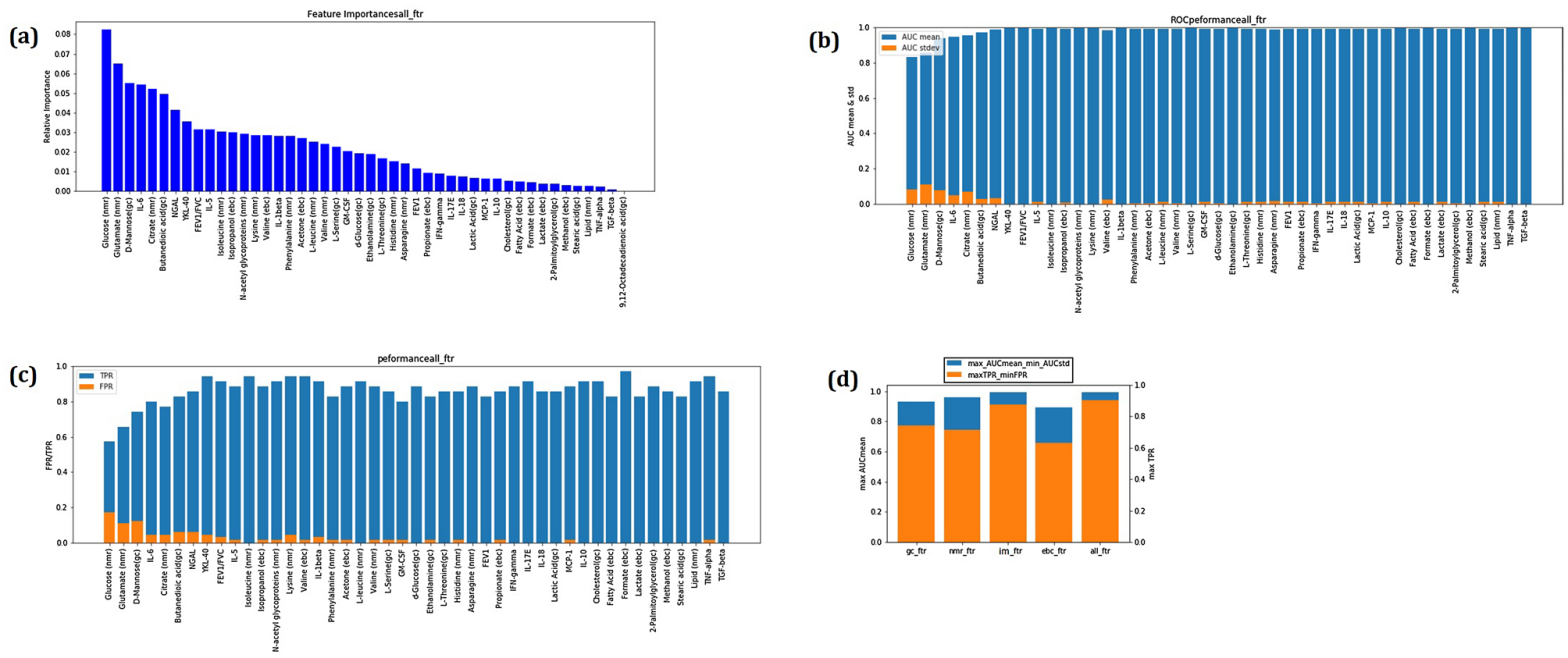
(**a**) Feature ranking obtained by mapping the feature importance scores (46 variables) which play an important role in predictive modelling using random forest (RF) classifier (number of trees = 50) which was built via tenfold cross-validation classifier and used to predict whether ACO can be effectively distinguished from asthma and COPD patients taken together. (**b**) The mean and standard deviations (stdev) in area under curve (AUC) of multivariate receiver operating characteristic (ROC) with the RF classifier of the most important features sequentially in a descending order of relative importance. (**c**) The corresponding true positive rate (TPR) and false positive rate (FPR) of multivariate ROC. (**d**) A combination of all platforms provides the highest mean AUC with least uncertainty and largest TPR with smallest FPR for robust classification of ACO from asthma and COPD. The analysis was performed using Scikit-learn package with Python 3.8 (https://scikit-learn.org/stable) (nmr)—depicts the altered metabolites detected in serum using NMR based metabolomics, (gc)—depicts the altered metabolites detected in serum using GC MS based metabolomics, (ebc)—depicts the altered metabolites detected in EBC using NMR based metabolomics, nmr_ftr—depicts the altered metabolites detected in serum using NMR based metabolomics, gc_ftr—depicts the altered metabolites detected in serum using GC MS based metabolomics, ebc_ftr—depicts the altered metabolites detected in EBC using NMR based metabolomics, im_ftr—depicts the altered immunological markers detected in serum, all_ftr—depicts the combination of features obtained from all the platforms.

Random forest (RF) classifier (number of trees = 50) was built via tenfold cross-validation and feature ranking obtained to map the feature importance scores which play an important role in predictive modelling, as shown in Fig. 5a. This provides an insight into the data as well as the model, and the basis for dimensionality reduction. Feature selection can improve the efficiency and effectiveness of a predictive model. A multivariate ROC analysis with the RF classifier was performed by adding the features sequentially in a descending order of relative importance (Fig. 5a). The mean and standard deviations (SDs) of ROC AUC and their corresponding true positive rates (TPR) and false positive rates (FPR) are presented in Fig. 5b,c, respectively.

Finally, a comparison of all platforms in terms of serum NMR metabolomic fingerprints, serum GC–MS fingerprints, EBC NMR metabolomic fingerprints and immunological mediator fingerprints using the same classifier was performed, both individually and taken together. It was observed that combination of all these platforms provide the highest mean AUC with least uncertainty and largest TPR with smallest FPR for robust classification of ACO from asthma and COPD (Fig. 5d).

## Discussion

EBC profiling using NMR metabolomics has increasingly gained popularity for the identification of metabolic phenotypes in various respiratory diseases. The present study discusses the metabolite content of EBC collected from subjects with ACO and their potential to discriminate ACO from asthma and COPD. NMR could unequivocally identify distinct metabolic signatures in EBC of patients with ACO. Furthermore, the method unambiguously recognizes metabolites responsible for between-group differences (asthma, COPD and healthy controls), thereby strongly suggesting identification of a set of promising unbiased biomarkers characterizing ACO.

Valine, an essential glucogenic amino acid, was found to be downregulated in ACO with respect to both asthma and COPD. Studies by other groups have shown that plasma levels of the branched chain amino acids (BCAAs) are significantly lower in patients with severe COPD than in controls^20,21^. Since BCAAs are utilized for muscle protein synthesis, their reduced levels appear to be consistent with muscle wasting and weight loss that are known to occur in advanced COPD^22^. It is, therefore, suggested that the significantly decreased level of valine in ACO reflects an enhanced metabolic demand. A similar trend in the expression of valine was also observed in serum of these subjects in our earlier study^18^.

The increased expression of lactate in EBC may be attributed to increased glycolysis due to an imbalance in oxygen supplement and demand, as explained by the “Warburg effect”. The concentration levels of lactate have been extensively studied in asthma^23,24^ and COPD^25,26^. Our observation, yet again, suggests an increased energy demand of ACO subjects. Also, a similar trend in lactate expression has been observed in serum of ACO subjects^19^.

Numerous groups have reported altered expressions of a common volatile organic compound (VOC), acetone in EBC of various respiratory diseases^16,21^. Montuschi et al. (2012) have indicated an increased expression of acetone in cystic fibrosis patients as compared with heathy controls^27^. This observation suggests increased lipid catabolism to meet the added energy requirements of the disease^28^. Our findings too indicate that the levels of acetone in EBC are significantly higher in ACO patients when compared to both, asthma and COPD.

Isopropanol or 2-propanol, yet another VOC, exhibited a similar trend in expression, as observed with acetone. It was found to be significantly upregulated in ACO. Elevated 2-propanol concentration in EBC might be due to bacterial metabolism and/or increased lipolysis and lipid peroxidation^29^. Endogenous formation of 2-propanol can occur in humans from reduction of acetone by liver alcohol dehydrogenase, mainly when increased levels of acetone and high NADH/ NAD + ratio occurs, as observed in ketosis^30^. Increased levels of 2-propanol concentration is also documented in COPD and cystic fibrosis cases^27,31^.

Methanol, another VOC, was also found to be significantly higher in ACO with respect to asthma, but lower in comparison to COPD. It is reported that increased methanol may be associated with airway inflammation^32^. Methanol, present in human breath^33^, is a breakdown product of formaldehyde, which is demonstrated to exacerbate airways inflammation in A549 alveolar and BEAS-2B bronchial cells^34^. Methanol levels in ACO may be linked to the heightened inflammatory status of the patients.

Altered levels of fatty acids in EBC are extensively reported^16,35–37^. Fatty acid levels in ACO are significantly dysregulated with an expression pattern similar to that of serum observed in these patients^19^. Short-chain fatty acids (SCFAs) regulate several leukocyte functions linked to the production of cytokines, eicosanoids, and chemokines and seem to affect leukocyte migration to the foci of inflammation^38^. Since they are also involved in energy requirement, their decrease might reflect the tight interplay between inflammation and energy production^36^. SCFAs can also originate from different pyruvate metabolism pathways^39^. Various studies on cell/animal models^40,41^ suggest that fatty acid residues can be harvested from lipids and may serve as energetic substrates in conditions of inhibited glycolysis and up-regulated β-oxidation^42^.

An increased expression of propionate was observed in EBC of ACO when compared with asthma and COPD. Given the role of propionate in the inhibition of cholesterol synthesis, its increase may also suggest the involvement of lipid metabolism. This finding is in agreement with the reports of several research groups, where increased lipolysis has been attributed to this trend^13,31^. Few groups have also suggested that increased propionate level could be the effect of drugs regularly administered to these patients^13^.

Elevated levels of formate in EBC are reported in patients with COPD as compared with healthy subjects^31,43^. These findings have been confirmed in a recent 1H-NMR spectroscopy study which shows that formate concentration in EBC is 2.5 higher in patients with emphysema due to α1-antitrypsin deficiency than in healthy subjects^13^. Formate levels were higher in ACO with respect to asthma but lower than that of COPD in the present study.

To make the findings more robust and reproducible, expression levels of the 8 metabolites identified were validated in a fresh cohort of subjects. A similar trend in expression was observed in the validation cohort. These findings are in accordance with our earlier serum metabolomic findings^18,19^. Dysregulation of a number of metabolites in EBC of ACO patients suggests that the energy and metabolic requirement in ACO is more severe when compared to COPD or asthma alone. This could be attributed to the higher disease severity associated with ACO. Also, multivariate ROC curves could discriminate ACO from the combined group of asthma and COPD with good sensitivity and specificity. Furthermore, the models generated from these multivariate ROC curves suggest that the combination of these metabolites provide a robust classification model which is better than using any one or two metabolites as a biomarker for differentiating ACO from both asthma and COPD taken together. In conclusion, NMR metabolomics using EBC as a biofluid holds potential to be explored as a platform for development of non-invasive biomarkers responsible for identification of ACO from a cohort of asthma and COPD.

**Figure 6:**
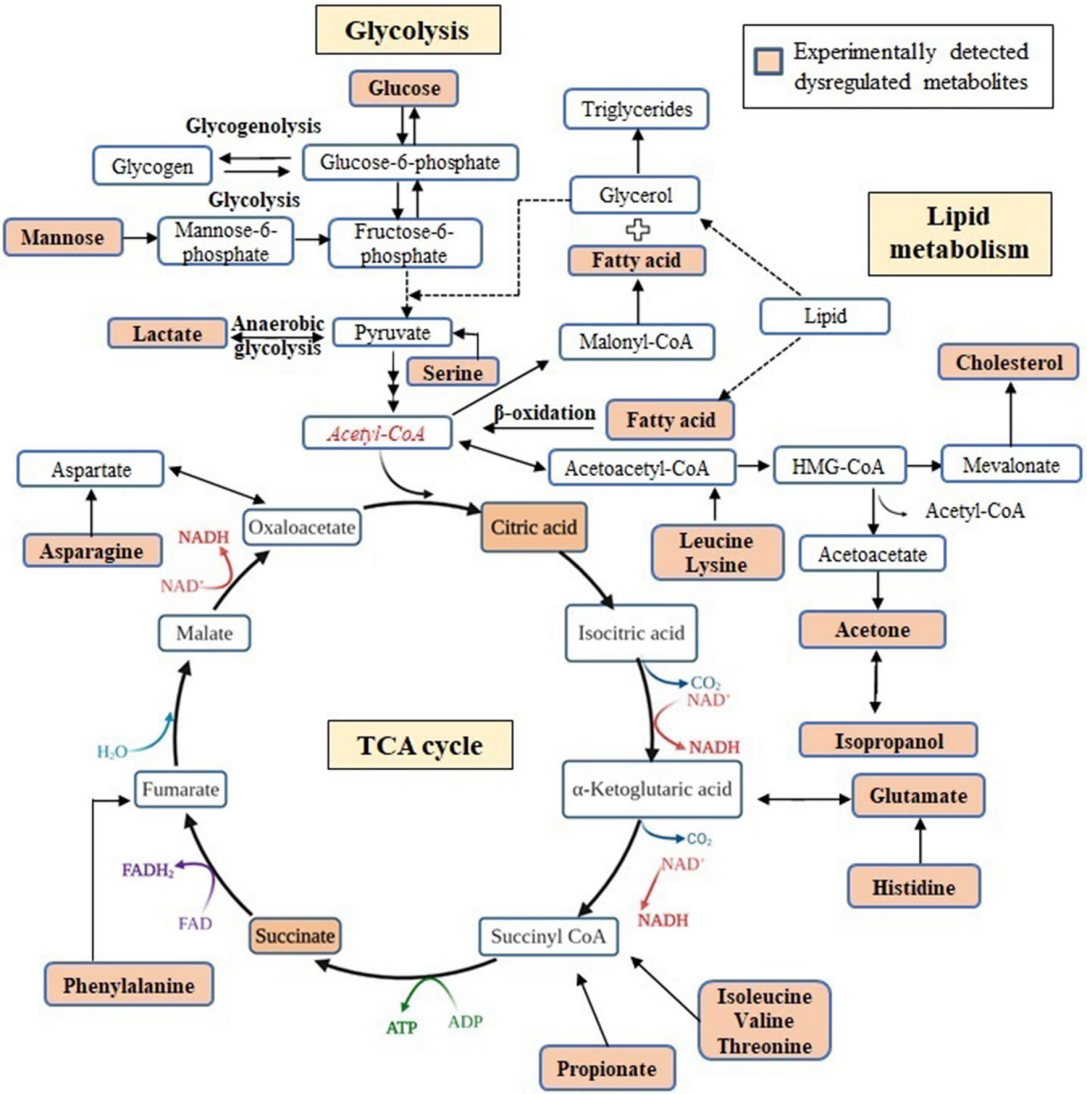
Proposed schematic diagram of the dysregulated biochemical pathways in ACO.

Earlier, metabolic and immunological signatures have been identified by our group in serum of these ACO subjects differentiating it from both asthma and COPD^19^. Here, a correlation heat map is generated using the 46 markers which comprise of a wide array of significantly altered metabolites, immunological mediators and clinical parameters from both the present and previous studies of our group. Using these variables, we have also attempted to use RF classifier to predict whether a combination of markers generated using various platforms is useful for optimum classification of ACO, and whether ACO can be effectively distinguished from asthma and COPD patients taken together. A random forest (RF) algorithm is an extremely reliable classifier and has become popular as a biomarker detection tool in various metabolomics studies. Using RF as a classifier has the following advantages: simple theory, fast speed, stable and insensitive to noise, little or no overfitting, and automatic compensation mechanism on biased sample numbers of groups^44–46^. It constructs an ensemble of decision trees, which is a combination of tree-structured predictors. Each tree is independently constructed using a bootstrap sample of the original data. This serves as the training data, which is used to build the classification model. Using predictive random forest algorithms on our present and earlier reported findings, it is concluded that a combination of 10 markers i.e. glucose, glutamate, D-mannose, IL-6, citrate, butanedioic acid, neutrophil gelatinase-associated lipocalin (NGAL), chitinase 3 like 1 (YKL-40), FEV1/FVC and IL-5 provide optimum classification of ACO. Based on these findings, a schematic representation of the various biochemical cycles found to be dysregulated in ACO subjects is shown in Fig. 6. It exhibits perturbation of key metabolism cycles such as tricarboxylic acid (TCA) cycle, glycolysis and lipid metabolism. This could possibly be responsible for the higher disease burden in ACO. Enhanced inflammatory response was also observed in patients with ACO. The dysregulated key immunological mediators hold promise in designing strategies for clinical management of ACO subjects in future.

There are several limitations associated with this study. First, the ACO subjects in this study are selected using GINA/GOLD and American Thoracic Society (ATS) roundtable diagnostic criteria; hence, the present findings should not be extended to patients diagnosed using other guidelines^7,47,48^. Second, here only South Asian male subjects are recruited; genetic, race and sex differences adjustment is necessary before generalization of our findings^49^. Third, the present findings are related to ACO patients without active exacerbations and respiratory infections. Alterations in metabolic signatures are likely in presence of lung infections and exacerbations^50–52^. Fourth, validation of the models in a fresh patient cohort could not be performed. However, we did include a fresh cohort of patients for each sub-group, i.e. asthma, COPD and ACO. Fifth, stage IV COPD patients could not be included since most of them reported with co-morbidities and exacerbations. However, we propose to conduct multi-centric studies to validate our findings. This will increase the sample size considerably and facilitate inclusion of a group of severe COPD patients for comparison with ACO. Finally, limited sensitivity of NMR spectroscopy as a metabolomics tool is well realized. Integration of NMR with complementary GC-qTOF and LC–MS/MS would increase metabolome coverage and improve data quality significantly. Our group is presently involved in using these approaches on the same patient cohort for complementary information.

EBC analysis has garnered a lot of interest in recent times. It is undoubtedly an advantageous technique, being a non-invasive, effortless, and painless method of respiratory biological material collection. However, it is a less explored biofluid as compared to serum, plasma, urine etc. and has certain limitations which require attention. The diversity of EBC itself has prevented it from achieving clinical applicability^12^. It is a dilute, complex solution of diverse molecules with different chemical stabilities. EBC is possibly not the best matrix for exploring VOCs and studies should be limited to non/semi volatile compounds. The low concentration of the dissolved molecules in EBC is one of its major limitations. The measurement of these molecules is limited by the sensitivity of most assays^53^. Standardization of biomarker analysis in EBC remains a challenge. High variability and low reproducibility in exhaled markers may be explained by differences in a large number of pre-collection, collection and post-collection conditions. Conditions and duration of storage may also affect the assayed concentrations of biomarkers. It is also important to note that despite EBC sampling established to be a method of sampling the airways that is generally acceptable to patients^12^, a few patients reporting to our clinic, especially those reporting with severe disease condition, found this technique challenging. This poses a question regarding the clinical utility of the method, though extensive use of this method in research is well recognized.

Comparison of efficacy and reproducibility of sampling between different devices requires further investigation. Further studies are also required to establish if different mediators are evenly distributed in the expired air and if collection of all water vapour expired would decrease variability of EBC data. Flow-dependency and the potential for oral/upper airway/salivary contamination needs to be explored^54^. Inter-individual differences in the generation of particles in EBC during inhalation may exist. As of yet no fully validated method for calculating dilution of respiratory droplets is available and the anatomic origin of biomarkers is not precisely known. Therefore, it can be said that even though EBC is a promising biofluid, there are a number of challenges which needs better understanding and standardizations before its potential can be fully explored.

Though biomarkers are known to positively impact patient care by permitting early detection of disease which increases therapeutic efficacy considerably, it is well recognized that very few omics-derived biomarkers have made their way to the clinic so far. Disease heterogeneity, complexity of the involved cellular processes, non-ideal study design, and limited methodological robustness are the major bottlenecks that are responsible for the huge gap between the number of omics-based biomarkers reported in literature and those introduced to the clinical set-up. Identification of a panel of markers highly specific to the disease with a perfect diagnostic potential of 100% sensitivity and specificity is desirable and remains a challenge. There is a need to establish the exact concentration and cut-off levels of candidate markers for their use in clinical settings.

In recent years, there has been an increasing interest in understanding the group of individuals having features of both asthma and COPD. As the complexity of ACO as a disease entity is gradually unraveled and better understood, a further revision in ACO definition would likely be required. It is envisaged that the findings from this exploratory study will motivate researchers and direct their attention towards unravelling the intricacies of ACO pathophysiology which remains to be elucidated.

## Material and methods

### Subject selection and sample collection

Patients reporting to the Institute of Pulmocare and Research (IPCR), a tertiary respiratory care clinic at Kolkata, India were considered for this study. Based on the clinical history, questionnaire data, pulmonary function tests (PFTs) and diagnosis by the clinicians, the subjects were divided into four groups: (i) patients with asthma (ii) patients with COPD (iii) patients with ACO and (iv) healthy controls with normal lung function. Informed consent was obtained from all participants. Approval was obtained from the Institutional Ethics Committee of IPCR, Kolkata prior to commencement of the study. The detailed inclusion and exclusion criteria are mentioned elsewhere^18,19^.

Asthma was diagnosed as per GINA (2014) guidelines^55^. Subjects with wheezing in the past 12 months and reversibility (increase in post-bronchodilator (post-BD) FEV1 or FVC ≥ 200 ml and ≥ 12% baseline change) or asthma diagnosed earlier were included. All patients enrolled were moderate or severe asthma cases. Diagnosis of COPD was according to the GOLD (2014) criterion^56^. Subjects with FEV1 to FVC ratio < 70% post BD were included. All COPD subjects included were moderate (Stage II) and severe COPD (Stage III) cases.

Joint guidelines of GINA and GOLD and ATS roundtable discussion^57–60^ was used to diagnose ACO. The main criteria considered were (i) persistent airflow limitation [post-BD FEV1/FVC < 0.70] in individuals 40 years of age or older (ii) tobacco smoking ≥ 10 pack-years and (iii) bronchodilator response (BDR) of > 400 ml in FEV1 or history of asthma before 40 years of age. The minor criteria taken into consideration were (i) history of allergic rhinitis or atopy (ii) BDR of FEV1 ≥ 200 ml and 12% change from baseline values on two or more visits (iii) peripheral blood eosinophil count ≥ 300 cells/μl. All major criteria and at least one minor criterion were considered for inclusion of ACO subjects. History of alpha-1 antitrypsin deficiency (AATD) in the family of COPD and ACO subjects was excluded. In order to avoid gender and smoking induced bias, only present/former male smokers were considered. All volunteers were also matched for age and BMI to minimize bias.

For comparison purposes, healthy age-matched male smokers were considered as controls. Patients with history of exacerbations and those who had received oral corticosteroid (OCS) treatment during the last three months were excluded. Patients with co-morbidities including metabolic disorders were also excluded. For this pilot metabolomic study, two independent patient cohorts, the discovery and validation group having the same exclusion and inclusion criteria were considered. The discovery phase patient cohort comprised of (i) asthma = 34 (ii) COPD = 30 (iii) ACO = 35 and (iv) controls = 33. The validation phase cohort consisted of (i) asthma = 32 (ii) COPD = 32 and (iii) ACO = 40 subjects.

EBC was collected in single-use disposable collection circuits using TURBO-DECCS 14 system (Medivac, Parma, Italy), according to the manufacturer’s instructions. All samples were collected based on the recommendations of the ATS/ERS Task force^12^. Patients were requested to be in resting condition for 30 min prior to collection. Smoking was not permitted for a minimum of 12 h. All samples were collected following a minimum of 12 h overnight fasting. Tidal breathing into the mouthpiece was advised for about 15–20 min till 2–3 ml EBC was collected. EBC collection vials were immediately sealed and stored at − 80 °C. During initial standardization of the collection process, two samples of EBC were collected from each subject for a total of 12 subjects (3 healthy controls, 3 asthma, 3 COPD and 3 ACO) within the same day (at time 0 h and 8 h). The spectral regions (excluding the water region) were integrated and normalized to the total spectrum area to avoid possible variation in metabolite concentrations. SD was calculated to check for natural variance and was found to be within ± 1.96, which according to the Bland–Altman test indicates good within-day repeatability^61^.

### Sample processing

Briefly, EBC samples were thawed and homogenized before performing the NMR experiments. A volume of 70 μl of D2O containing 1 mM TSP and 3 mM sodium azide was added to 630 μl of thawed condensate, thus making 700 μl of total volume and centrifuged at 8000 rpm for 5 min. The samples were transferred to 5 mm NMR tubes and subjected to NMR analysis.

### NMR measurements

1D NMR spectra of EBC samples were acquired, as discussed elsewhere^31,62,63^. Briefly, 1D spectra were recorded on a Bruker AVANCE NEO 800 MHz spectrometer equipped with TCI CryoProbe operating at a frequency of 800 MHz (1H) and at a probe temperature of 27 °C. The water resonance was suppressed by using the excitation sculpting pulse sequence, zgesgp according to the manufacturer’s instructions. In the present study, the carrier frequency (01) value was set at the water resonance, the relaxation delay was 3.5 s, acquisition time 0.85 s, the spectral width was 9615 Hz, the time domain was 16 K and the number of transients was 256. This resulted in a total acquisition time of about 18 min per sample. For every sample, the probe was perfectly tuned and the 90 degree pulse width determined. For processing, a shifted sine bell (SSB) of 2 Hz was applied before Fourier transformation and a real spectrum size of 16 K used.

The resulting spectra were phased and baseline corrected offline using MestReNova version 7.1.0 (Mestrelab Research, Santiago de Compostela, Spain) software. TSP served as the chemical shift reference point (δ = 0.00 ppm) and a concentration standard for all samples. Each metabolite was identified from earlier published literature and cross verified with the HMDB 3.6 and 2D NMR experiments like correlation spectroscopy (COSY) and total correlation spectroscopy (TOCSY).

Quality control (QC) samples were prepared to monitor the analytical variability and reproducibility of NMR data acquisition over time. Equal volume of EBC (100 µl) was aliquoted from all subjects (discovery and validation cohort) and pooled together to prepare the QC samples. A total of 4 QC samples were included per day with each sample introduced at the beginning, after every 10 samples, and at the end of each day, respectively of NMR experimentation (~ 25 samples per day). CVs for each of the 18 consistently identified and quantified metabolites were calculated based on the QC samples (~ 40) and was found to be < 15%. In addition, pH of each sample (including QC samples) was checked and adjusted prior to each run.

### Data pre-processing

Multivariate analysis was performed on the spectral region of 0.5–4.0 ppm (excluding residual water signal: 4.0–6.0 ppm). This region was bucketed/binned into various integrated segments of equal frequency window (0.03 ppm) using MestReNova. The binned data matrix was normalized and scaled to the working region of 0.5 to 4.0 ppm. Normalization (by sum) was performed on the binned data sets to compensate for the variation in concentration between the samples and to represent each data point as a fraction of the total integral value of the spectra. Following normalization, unit variance scaling was performed using Metaboanalyst 4.0. Data transformation and scaling are two different strategies to make features more comparable. The preprocessed data was subjected to MVA using SIMCA 13.0.2 (Umetrics, Sweden)^64^.

### Multivariate analysis and statistical model validation

Various multivariate statistical approaches exist to understand the complex structures of metabolomic data. Supervised classification models including PLS-DA and OPLS-DA were generated using SIMCA 13.0.2 (Umetrics, Sweden). A coefficient of variation plot was used to represent differences in the metabolite concentration between the groups. The parameters including R2, Q2, and CV-ANOVA score were used to detect robustness of the OPLS-DA model. The most significantly altered bins (corresponding to dysregulated metabolites) were identified based on VIP score with a threshold of VIP ≥ 1 and S-line plot with correlation value r ≥ 0.6.

### Univariate statistical analysis of selected metabolites

Following constant sum normalization using MestReNova, the selected altered metabolite signals in the discovery cohort were extracted and subjected to spectral integration. Statistical significance of the mean integral values for the corresponding metabolites between the groups was obtained using one way ANOVA (Dunnett’s post hoc test) or Kruskal–Wallis test (Dunn’s post hoc test), whichever applicable (GraphPad Prism version 7.00 for Windows, GraphPad Software, San Diego, CA, USA). The peaks identified in the chemical shift region 6.0–9.0 ppm (aromatic region) were also subjected to one way ANOVA (Dunnett’s post hoc test) or Kruskal–Wallis test (Dunn’s post hoc test), as applicable. Statistical significance was considered to be p < 0.05. Fold change analysis was also performed for ACO vs. asthma and ACO vs. COPD.

The most significantly altered metabolites identified in the discovery phase were further validated by integrating the spectra for quantitative measurements in a fresh cohort of subjects (validation cohort) using the same univariate techniques.

### Multivariate ROC curve correlation analysis and predictive modelling

Multivariate ROC was performed on all significantly altered EBC metabolites and the AUC calculated (Metaboanlyst 4.0) using classification method support vector machine (SVM) and feature ranking as SVM built-in.

Our earlier studies using GC–MS metabolomics indicated 11 metabolites [serine, threonine, ethanolamine, glucose, cholesterol, 2-palmitoylglycerol, stearic acid, lactic acid, linoleic acid, D-mannose and succinic acid] to be significantly altered in serum of ACO patients as compared with asthma and COPD^19^. NMR based metabolomics evidenced dysregulation of 12 metabolites including lipids, isoleucine, N-acetylglycoproteins (NAG), valine, glutamate, citric acid, glucose, L-leucine, lysine, asparagine, phenylalanine and histidine in ACO patients^18^. Also, 13 immunological mediators including tumor necrosis factor alpha (TNF-α), IL-1β, IL-17E, granulocyte macrophage-colony stimulating factor (GM-CSF), IL-18, NGAL, IL-5, IL-10, monocyte chemoattractant protein- 1 (MCP-1), YKL-40, interferon gamma (IFN-γ), IL-6 and transforming growth factor (TGF-β) showed distinct expression patterns in ACO^19^.

A correlation heat map was generated using a total of 46 variables dysregulated in ACO. The wide array of significantly altered metabolites, immunological mediators and clinical parameters consisted of 8 EBC markers identified in the present study, 2 lung function parameters, and 36 serum markers generated from our previous studies. Pearson’s correlation coefficients were calculated between all the features using Scikit-learn package with Python 3.8. Using these 46 variables, RF classifier^65^ was used to predict whether ACO can be effectively distinguished from asthma and COPD patients taken together. To develop this binary classifier, ACO was defined as one group and asthma and COPD, taken together, as the other.

### Ethics approval

All procedures performed in the study involving human participants were done in accordance with the ethical standards of the institutional research committee and with the 1964 Helsinki declaration and its later amendments.

### Consent to participate

Informed consent was obtained from all individual participants included in the study.

## Supplementary Information

Supplementary Information.

## Data Availability

The datasets used and/or analysed during the current study are available from the corresponding author on reasonable request.
