# Supplementary material for: Global metabolome profiling of exhaled breath condensates in male smokers with asthma COPD overlap and prediction of the disease
Source: Sci Rep. 2021 Aug 17;11:16664. doi: 10.1038/s41598-021-96128-7 (PMC8370999; doi:10.1038/s41598-021-96128-7)
Supplement: Supplementary file 1 — Supplementary Information. [file 41598_2021_96128_MOESM1_ESM.pdf]

Supplementary Materials

Title: Global metabolome profiling of exhaled breath condensates in male smokers with asthma COPD overlap and prediction of the disease

Authors: Nilanjana Ghosh[a], Priyanka Choudhury[a], Mamata Joshi[b], Parthasarathi Bhattacharyya[c], Sushmita Roychowdhury[d], Rintu Banerjee[e], Koel Chaudhury[a*]

Affiliations:

[a]School of Medical Science and Technology, Indian Institute of Technology Kharagpur (India)

[b]National Facility for High-field NMR, Tata Institute of Fundamental Research, Mumbai (India)

[c]Institute of Pulmocare and Research Kolkata (India)

[d]Apollo Gleneagles Hospitals Kolkata (India)

[e]Department of Agricultural and Food Engineering, Indian Institute of Technology Kharagpur (India)

Corresponding author: Dr. Koel Chaudhury, Ph.D

Professor, School of Medical Science & Technology (SMST)

Indian Institute of Technology Kharagpur

Kharagpur - 721302, India

Email for all correspondence: koel@smst.iitkgp.ac.in; koeliitkgp@gmail.com

Ph: +913222-283572

Multivariate analysis of validation phase data

Multivariate statistical analysis (SIMCA 13.0.2, Umetrics, Sweden) was performed on the validation phase data to strengthen our discovery phase findings. The supervised classification models including partial least squares discriminant analysis (PLS-DA) and orthogonal partial least squares discriminant analysis (OPLS-DA) were generated. OPLS-DA is an extension of the PLS-DA method which filters out the variability that is not directly related to class separation. Coefficient of variation was used to represent differences in metabolite concentration between the groups. Parameters including R2, Q2, and analysis of variance testing of cross validated predictive residuals (CV-ANOVA) score were used to detect robustness of the OPLS-DA model

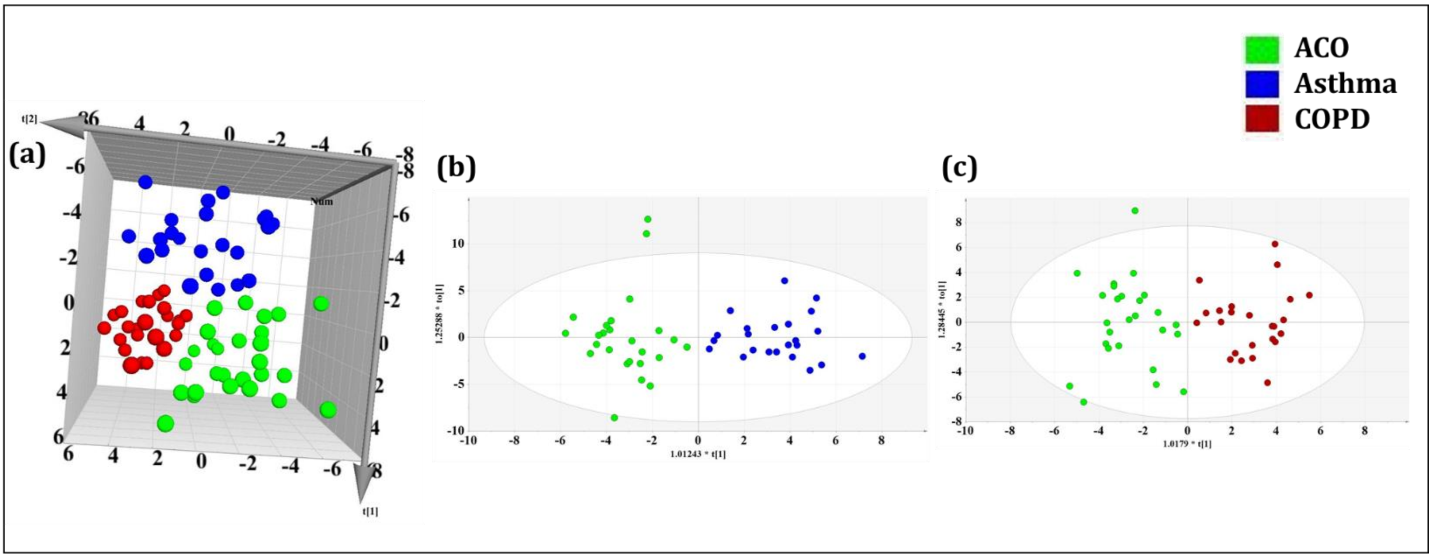

Supplementary Fig. 1. (a) Partial least squares discriminant analysis (PLS-DA) showing optimized discrimination between asthma, COPD and ACO (R2Y= 0.649 and Q2=0.513, CV-ANOVA= 2.14E-23). Orthogonal projections to latent structures discriminant analysis (OPLS-DA) shows optimized discrimination between (b) ACO and asthma (R2Y = 0.832 and Q2 = 0.705, CV-ANOVA score p = 6.35E-11) and (c) ACO and COPD (R2Y = 0.811 and Q2 = 0.537, CV-ANOVA score p = 4.81E-17). COPD- Chronic obstructive pulmonary disease, ACO-asthma COPD overlap

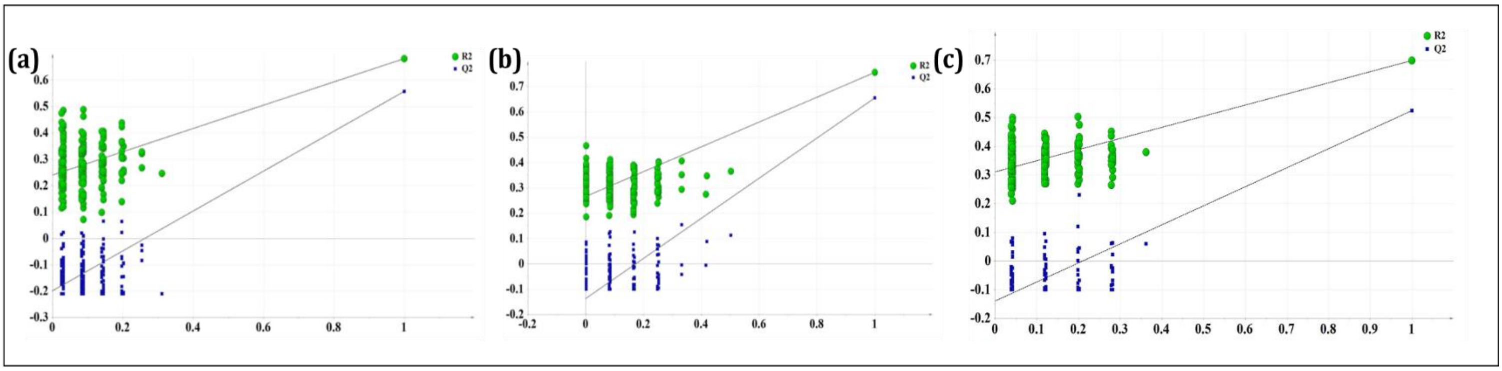

Supplementary Fig. 2. Response permutation test (n=200) to estimate the statistical significance of the supervised models. The R2 and Q2 values on the extreme right-hand side of the plot are of the true model, whereas the permutated model parameters are represented on the left-hand side of the plot. The correlation coefficients between true and permutated models represent the X axis. The true class has a correlation of 1.0 with itself. The true model parameters in the validation test exhibited higher values than those of the permutated models. (a) ACO, Asthma, COPD R2=(0.0,0.24), Q2=(0.0,-0.199) (b) ACO vs. Asthma R2=(0.0,0.266), Q2=(0.0,-0.137) (c) ACO vs. COPD R2=(0.0,0.311), Q2=(0.0,-0.139)
